# Spider-Venom Peptides: Structure, Bioactivity, Strategy, and Research Applications

**DOI:** 10.3390/molecules29010035

**Published:** 2023-12-20

**Authors:** Ruiyin Guo, Gang Guo, Aili Wang, Gaochi Xu, Ren Lai, Hui Jin

**Affiliations:** 1 Center for Evolution and Conservation Biology, Southern Marine Science and Engineering Guangdong Laboratory (Guangzhou), Guangzhou 511458, China; guory@gmlab.ac.cn (R.G.);; 2 The Third Affiliated Hospital of Kunming Medical University (Yunnan Cancer Hospital), Kunming 650118, China; 13888480101@139.com; 3 Key Laboratory of Bioactive Peptides of Yunnan Province, KIZ-CUHK Joint Laboratory of Bioresources and Molecular Research in Common Diseases, National Resource Center for Non-Human Primates, Kunming-Primate Research Center, National Research Facility for Phenotypic & Genetic Analysis of Model Animals (Primate Facility), Sino-African Joint Research Center and Engineering Laboratory of Peptides, Kunming Institute of Zoology, Kunming 650107, China

**Keywords:** spider-venom peptides, structure, bioactivity, survival strategy, therapeutics

## Abstract

Spiders (Araneae), having thrived for over 300 million years, exhibit remarkable diversity, with 47,000 described species and an estimated 150,000 species in existence. Evolving with intricate venom, spiders are nature’s skilled predators. While only a small fraction of spiders pose a threat to humans, their venoms contain complex compounds, holding promise as drug leads. Spider venoms primarily serve to immobilize prey, achieved through neurotoxins targeting ion channels. Peptides constitute a major part of these venoms, displaying diverse pharmacological activities, and making them appealing for drug development. Moreover, spider-venom peptides have emerged as valuable tools for exploring human disease mechanisms. This review focuses on the roles of spider-venom peptides in spider survival strategies and their dual significance as pharmaceutical research tools. By integrating recent discoveries, it provides a comprehensive overview of these peptides, their targets, bioactivities, and their relevance in spider survival and medical research.

## 1. Introduction

Spiders (Araneae), with over 47,000 described species and an estimated 150,000 extant species, have been prominent on Earth for over 300 million years. Their remarkable evolutionary journey equipped them with venom components to paralyze and kill their prey, which is often many times larger than themselves, rendering them the most successful venomous animals in evolution. Due to the strong toxicity of spider venom, it can produce several negative effects on human health, such as pain, swelling, diaphoresis, hypertension, patchy paralysis around the site of the bitten area, and, in some cases, may cause death [1]. While spider venom’s strong toxicity can harm human health, a negligible fraction of extant spider species (i.e., only 0.5%) pose a risk to human health [2]. It is these small groups of species that secrete many pharmacologically complex venoms, bringing humans a rich source that provides lead compounds or drug candidates for human diseases.

The primary purpose of spiders’ venom lies in their survival strategies, such as predation, defense, and competitor deterrence. Within the arachnid family, spiders and scorpions have emerged as prominent producers of venom through specialized venom glands. Except for Symphytognathidae, Uloboridae, and certain Mesothelae species, the majority of spiders are equipped with chelicerae harboring venom glands [3]. These venoms predominantly contain neurotoxins, each selectively targeting specific ion channels [4]. The rapid paralysis induced by neurotoxins can be a very effective way for spiders to immobilize their prey [5].

Spider venoms are complex mixtures of low molecular weight organic components, proteins, polypeptides, neurotoxins, nucleic acids, free amino acids, inorganic salts, and monoamines [6]. The functionally most important components of spider venoms are peptides with different pharmaceutical activities, including antibacterial, antifungal, anticancer, and analgesic effects. Some of these peptides are directed against a broad range of pharmaceutical targets, rendering them valuable resources for potential therapeutic candidates [7]. Integrative analyses of proteomics and the cDNA library show that the venoms of Australian funnel-web spiders (*Atrax robustus and Hadronyche versuta*) comprise more than 1000 peptides with a molecular weight ranging from 2 to 8 kDa [7,8]. Hence, we conducted a thorough review of the advancements and potential of spider-venom peptides, encompassing their chemical structure, biological activity, mechanisms of action, role in survival, and both direct and indirect applications in drug development [9].

Despite the limited characterization of spider-venom peptides in pharmacological studies, the range of known biological activities is impressive [10]. Most characterized peptides exhibit high affinity for specific molecular targets and demonstrate selectivity for particular receptors, including various ion channels and their subtypes, as discussed below [7]. Notably, disulfide-rich spider peptides exhibit extraordinary pharmacological activities by acting as modulators of mechanosensitive channels, acid-sensing ion channels (ASICs), calcium-activated potassium (KCa) channels, voltage-gated potassium (Kv) channels, voltage-gated sodium (Nav) channels, voltage-gated calcium (Cav) channels, glutamate receptors, and glutamate transporters [11,12,13]. In addition to their potential as drug candidates, spider-venom peptides also serve as valuable tools in the research of electrophysiological, pharmacological, and structural aspects of ion channels, as well as in modeling ion channel-related diseases. A newly detected neurotoxin, known as µ-TRTX-Hl1a, containing 39 amino acid residues, has been uncovered in the venom of the *H. lividum* spider. Meng et al. (2016) revealed that µ-TRTX-Hl1a inhibits the sodium channel subtype Nav1.8, and may offer significant utility for physiology research focused on NaV1.8 [5]. Piezo is a mechanosensitive cation channel responsible for the stretch-mediated Ca^2+^ and Na^+^ influx in multiple types of cells. Intraperitoneal injection of a Piezo1 channel blocker, GsMTx4, ameliorated experimental pulmonary hypertension in mice. Upregulation of the mechanosensitive channel Piezo1 may play a critical pathogenic role in the development of pulmonary vascular remodeling in pulmonary arterial hypertension and pulmonary hypertension [14].

Many reviews have summarized the biochemical, pharmacological complexity and therapeutic potential of the spider-venom peptides, but few focus on biological and pharmacological research. Additionally, the significance of toxins as a vital defense strategy for the spider’s own survival has been scarcely discussed. In this review, based on adding to the recent advance in spider-venom peptides, we will comprehensively summarize and review various aspects of spider-venom peptides, including their structure, diversity in biological activity, impact on spider survival, potential in drug development, and their utility as tools in biological and pharmacological research.

## 2. Structure

Spider venoms typically contain various forms of enzymes, polyamines, salts, and peptides. Unlike the venoms of many other animals, which comprise a mix of unrelated peptides, often from different gene superfamilies and in varying proportions, spider-venom peptides are remarkably specific. They predominantly feature a single molecular scaffold of ancient evolutionary origin as their main venom components [3]. The research on the structure of spider-venom peptides can deepen our understanding of toxin activity and functional targets, and also potentially help with drug development and disease treatment.

**Figure 1:**
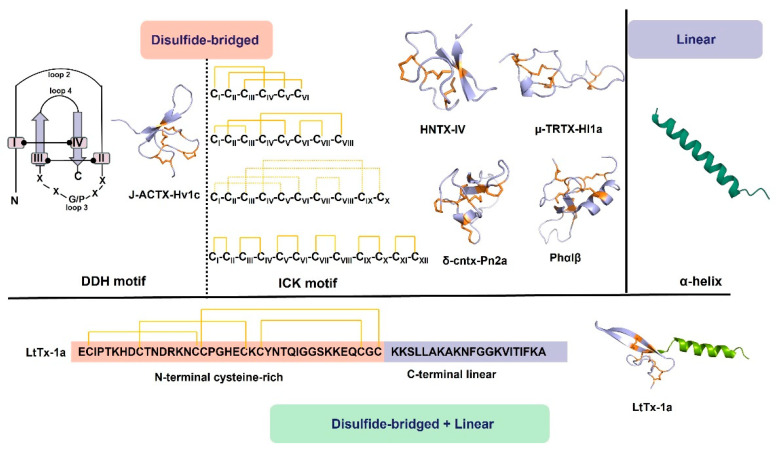
Structures of the spider-venom peptides. The major peptides of spider venoms are CRPs with multiple disulfide bonds, including the ICK motif and DDH motif. Solid orange lines indicate experimentally determined disulfide bonds and dotted orange lines indicate predicted disulfide bonds. In addition, linear peptides mainly assume amphipathic α-helices in the membrane-mimicking environment, which are also common in spider venom. In addition, some spider venoms consist of an N-terminal inhibitor ICK β domain and a C-terminal linear cationic domain, e.g., LtTx-1a [35]. J-ACTX-Hv1c (UniProt: P82228), HNTX-IV (UniProt: D2Y232), µ-TRTX-Hl1a (AlphaFold prediction), δ-cntx-Pn2a (UniProt: P29425), Phα1β (UniProt: P81792), LtTx-1a (UniProt: B3EWF2). Disulfide bridges are shown in orange.

Disulfide-bridged peptides in spider toxins adopt two primary structural motifs, the first motif is the inhibitory cystine knot (ICK) (Figure 1), which is prevalent among known spider peptide toxins. This peptide is characterized by the classical consensus sequence CIX_2–7_-CIIX_3–11_-CIIIX_0–7_-CIVX_1–17_-CVX_1–19_-CVI and characteristic CI-CIV, CII-CV, and CIII-CVI disulfide bonds (the cysteine residues are labeled I–VI in order from the N- to the C-terminus), where X represents amino acids of varying lengths [10,15,16,17]. Typically, ICK contains three disulfide bonds in a sequence, with two disulfides forming the loop and the third disulfide penetrating through the loop [18]. This structure possesses exceptional stability, rendering peptides with the ICK motif, particularly those with additional disulfide bonds, highly resistant to denaturation and proteolysis. The second motif is the disulfide-directed β-hairpin (DDH), where its main sequence is CX_5−19_CX_2_ (G or P) X_2_CX_6−19_C [19]. Unlike the ICK motif, the DDH motif lacks cysteine knots and consists of an antiparallel β-hairpin stabilized by two disulfide bonds (Figure 1), which constitute the majority of the hydrophobic core. Consequently, loop one no longer requires a connection to the N-terminal cysteine as in the ICK motif, and loop three typically contains five amino acid residues, with a Gly or Pro in the middle to ensure the stability of the first β-strand [5,20].

Furthermore, spider venoms also contain linear peptides (Figure 1), which lack disulfide bonds. These linear peptides primarily exhibit cytolytic properties and possess antimicrobial and antitumor activities [21,22,23,24]. Consequently, linear peptides are categorized as a distinct class of spider-venom components. In a membrane-mimicking environment, linear spider-venom peptides predominantly adopt amphipathic α-helices [25,26,27]. The α-helical structure is mainly composed of hydrophilic amino acid residues on one side and hydrophobic amino acid residues on the other side. The positive charge on the hydrophilic face interacts with the negatively charged prokaryotic membrane, while the hydrophobic side attaches to the lipid bilayer, leading to its disruption. [25,28]. With the change of pH or polysialic acid, α-helical linear peptides may undergo structure changes, adopting configurations such as helix–hinge–helix, disordered, or β-sheet structures [29,30]. Additionally, mutations, fatty acid, or amidation modifications in the peptide sequence may change the activity of linear peptides [31,32,33]. Studies have found that the antibacterial activity of linear peptides in spider toxins is attenuated in the presence of Glu residues on the hydrophilic surface of the α-helical structure [22].

Interestingly, some spider venoms exhibit a unique structural composition, featuring an N-terminal inhibitor cystine knot (ICK) β domain, as seen in neurotoxins, along with a C-terminal linear cationic domain, akin to cytolytic peptides [34,35]. These peptides exhibit a distinctive structural arrangement. In an aqueous solution, the C-terminal segment is very flexible, whereas the knottin domain is very rigid. In a membrane-mimicking environment, the C-terminal domain assumes a stable amphipathic α-helix. Thus, the α-helical structure effectively tethers the peptide to the membrane and serves as a membrane anchoring device [34,35].

## 3. Bioactivity

### 3.1. Antimicrobial Activity

**Figure 2:**
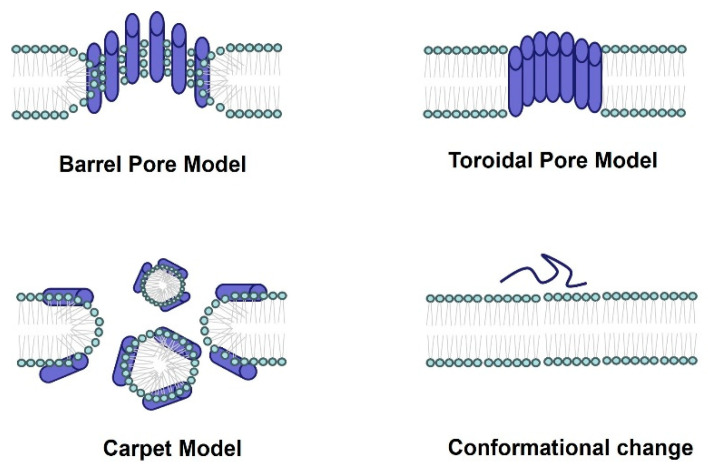
The antimicrobial mechanism of spider-venom peptides. Multiple models elucidating the membranolytic activity and antimicrobial mechanism of spider-venom peptides. In the barrel pore model, spider-venom peptides directly insert into the membrane, forming a pore where the hydrophilic regions of the alpha helices face the center of the pore. The toroidal pore model resembles the barrel pore model, with pore-forming peptides interacting with lipid head groups. The carpet model depicts spider-venom peptides acting on membranes, leading to micelle formation and the generation of membrane holes. Additionally, the conformational changes in spider-venom peptides contribute to alterations in their antimicrobial activity.

With the growing threat of antibiotic resistance, the demand for new antimicrobial drugs has become urgent. Spider-venom peptides are notably characterized by their antimicrobial activity [23] (Table 1). These peptides exert their antimicrobial effects through membrane permeabilization or disruption. Possible mechanisms of pore formation include the barrel pore model, toroidal pore model, carpet model, and conformational change (Figure 2). For instance, Juruin, derived from the spider venom of *Avicularia juruensis*, is a 38-residue cationic peptide containing three disulfide bonds arranged in an inhibitory ICK motif and a valine amidated at the C-terminus. It has shown potential antimicrobial activity against various human clinical isolates, including *Candida krusei*, *C. glabrata*, *C. albicans* sp., *C. parapsilosis*, *C. tropicalis*, *C. guilliermondii*, and *Aspergillus niger* [36]. An analysis of the amino acid sequence reveals a high degree of similarity between Juruin and insecticidal peptides found in the theraposid spiders *Selenocosmia huwena*, *Chilobrachys jingzhao*, and *Haplopelma schmidti*, indicating that perhaps they may share similar antimicrobial activity [36]. Peptides Lycocitin 1 and 2 (both are C-terminally amidated and positively charged) from the spider venom of *Lycosa singoriensis* have been found to inhibit the growth of Gram-positive (*S. aureus, B. subtilis*) and Gram-negative (*E. coli, P. aeruginosa*) bacteria, and fungi (*Candida albicans*) [37]. *Lachesana tarabaevi* produce seven short linear cationic amphipathic α-helical peptides named Ltc 1, Ltc 2a, Ltc 3a, Ltc 3b, Ltc 4a, Ltc 4b, and Ltc 5. These peptides have demonstrated significant antimicrobial effects against Gram-positive (*A. globiformis* and *B. subtilis*) and Gram-negative (*E. coli* and *P. aeruginosa*) bacterial strains in vitro.

### 3.2. Anticancer Activity

Cancer, a life-threatening disease with high mortality rates, poses a significant threat to global health [46,47]. Over the past few decades, significant advances in the treatment of cancer have been focused on chemotherapy, radiation therapy, immunotherapy, and surgery. Nevertheless, the major problems of these conventional therapies were the relatively low success rate, serious side effects, and drug resistance [48,49,50]. Consequently, there is a growing need for more effective and less toxic treatments for cancer patients.

**Figure 3:**
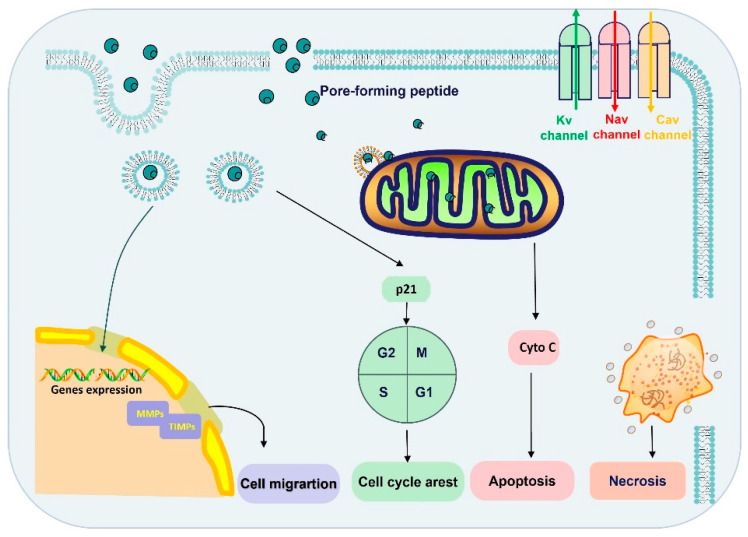
The anticancer mechanism of spider-venom peptides. The anticancer mechanisms of spider peptides include pore formation and blockade of ion channels (Nav, Kv, and Cav channels). Ion channel overexpression and dysfunction are associated with tumor progression, invasion, and metastasis. On the other hand, spider-venom peptides penetrated into cytoplasm by direct membrane rupture or endocytosis, which induced inactivation of mitochondria and necrosis. Moreover, spider-venom peptides can bind with the mitochondria membrane and promote a mitochondria-mediated death pathway to induce tumor cell apoptosis. In addition, the spider-venom peptides regulate p21 protein expression to arrest the cell cycle and the level of MMP/TIMP changes also restricts migration. Therefore, spider-venom peptides regulate tumor cells through a variety of ways to play an anticancer role. Cyto C: Cytochrome C.

Recent studies have shown that spider-venom peptides exhibit potential anticancer activity (Table 2). As illustrated in Figure 3, peptides have shown the ability to suppress cancer by disrupting tumor cell membranes, inhibiting cancer cell growth, inducing necrosis, impeding cell migration, promoting apoptosis, modulating ion channels (Nav, Kv, and Cav channels), and forming pores in tumor cells [51]. For instance, Brachyin, a neurotoxin isolated from the venom of the spider Brachypelma albopilosum, has demonstrated significant inhibitory effects on cell proliferation in various cancer cell lines, including C8166, Molt-4, A549, BIU-87, T24, and Calu-6, with IC50 values ranging from 1.5 to 24 µg/mL. Notably, this peptide exhibited more potential inhibition against C8166 compared to other cell lines [21]. Lycosin-I, a 24-amino acid peptide from the venom of Lycosa singorensis, has demonstrated potent anti-tumor activity in vitro and in vivo [24]. It has been observed that Lycosin-I penetrates cell membranes to activate the mitochondrial apoptotic pathway, sensitizing cancer cells to apoptosis, and upregulates p27 to inhibit cell proliferation [24]. As reported, the amphiphilic-helix conformation of Lycosin-I was induced by lipid membranes and subsequently stably aggregated on the bilayer, which were believed to be crucial for cell penetration [52].

### 3.3. Insecticidal Peptides

Spider venoms represent a bountiful source of disulfide-rich insecticidal peptides that have evolved over millions of years to precisely target a diverse array of receptors and ion channels within the insect nervous system. Most of these peptides have a unique disulfide bond arrangement that provides extreme resistance to proteases. Consequently, these peptides are highly stable in the insect gut and hemolymph, making them potential insecticides.

**Figure 4:**
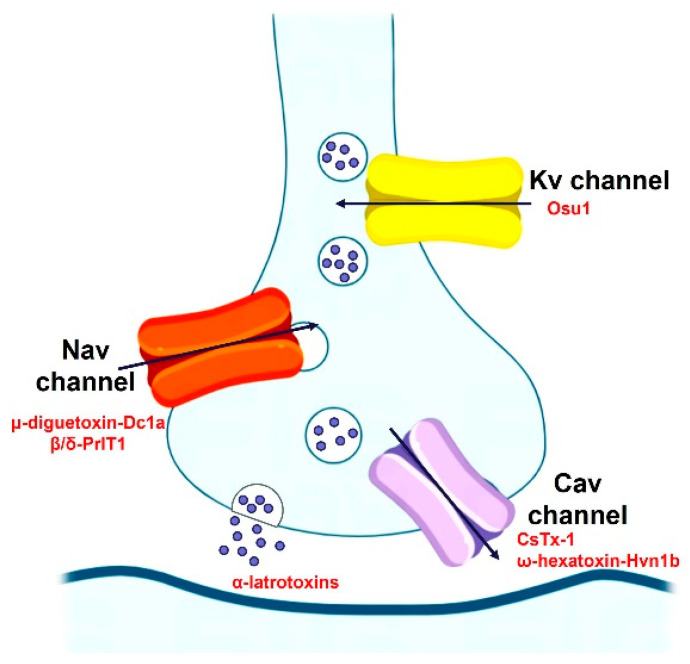
The insecticidal mechanism of spider-venom peptides.

Most Spider-venom peptides target synapses in the insect central nervous system to kill them. Spider venoms enhance the penetration of peptide and protein neurotoxins into their molecular targets by degrading the myelin sheath around axons and the extracellular matrix of the synaptic cleft. For example, the α-latrotoxin binds to specific receptors on presynaptic nerve terminals, which enables it to subsequently insert into the nerve terminal membrane to form a nonselective cation channel, which cause massive neurotransmitter release by promoting synaptic vesicle exocytosis. Additionally, spider-venom peptides modulate ion channels of the insect central nervous system, such as the Nav channel (μ-diguetoxin-Dc1a, β/δ-PrIT1), Kv channel (Osu1), and Cav channel (CsTx-1, ω-hexatoxin-Hvn1b), acting together in a synergistic manner to maximize the overall effect of the venom on prey (Figure 4). For instance, μ-diguetoxin-Dc1a (Dc1a), as detailed in Table 3, stands out as a formidable and selective insecticidal spider peptide (ISP). Its mode of action involves the blockade of insect voltage-gated sodium (Nav) channels. Dc1a has proven efficacious in exterminating German cockroaches, albeit American cockroaches remain insensitive to its effects [58]. Moreover, studies have revealed that the unrelated ISP μ-theraphotoxin-Ae1a (Ae1a) also engages the identical binding site on insect Nav channels to Dc1a. Its susceptibility to the toxin is attributed to inherent sequence variations in the S1–S2 loop of domain II [59]. The potency and specificity of ISPs such as Dc1a and Ae1a highlight the possibility of selectively targeting certain pest species without harming beneficial insects or vertebrates, including humans, thereby reducing environmental pollution.

Spider-venom peptides kill insects by targeting a diverse array of receptors and ion channels in the insect nervous system, such as the Nav channel (μ-diguetoxin-Dc1a, β/δ-PrIT1), Kv channel (Osu1), and Cav channel (CsTx-1, ω-hexatoxin-Hvn1b).

## 4. Venom Peptides’ Roles in Spider Survival

**Figure 5:**
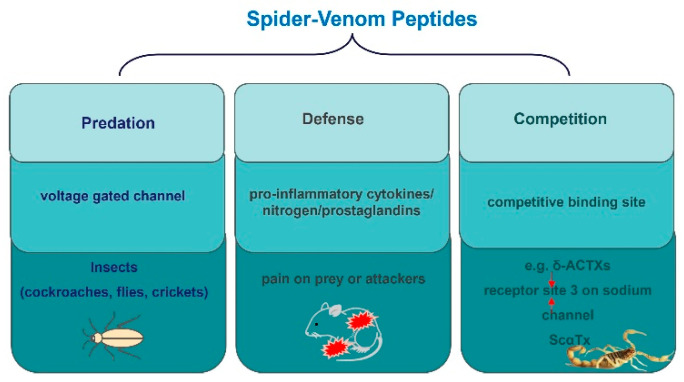
The role of peptides in the survival of spiders.

Spiders employ venom as a multifaceted survival strategy, which encompasses predation, defense, and competition (Figure 5) [70,78,79,80]. The insecticidal function of spider toxins is primarily achieved by acting on voltage-gated ion channels in insect neurons [81,82,83]. For instance, μ-NPTX-Nc1a from Nephila clavate effectively inhibits the Nav and Kv channels in cockroach DUM neurons, leading to facial paralysis and eventual death in cockroaches, with a determined LD50 of 135 pmol/g [24,79,84]. Similarly, δ-HXTX-Ar1a from *Atrax robustus* demonstrates inhibitory activity on the Nav channels in cockroaches and flies and has insecticidal effects on sheep blowflies, with a median paralytic dose (PD_50_) of 319 pmol/g [79,83]. Furthermore, ω-Tbo-IT1, originating from the Tibellus oblongus spider, exhibits concentration-dependent inhibition of Ca^2+^ channels in Periplaneta americana cockroach neurons, with IC50 values of 40 ± 10 nM [70]. Conversely, Tbo-IT2 exhibits insecticidal activity on housefly larvae (LD100 200 μg/g), but remains inactive on expressed neuronal receptors and ion channels, including sodium, potassium, calcium, proton-activated ASIC1 channels, and TRP channels [80]. The target of the Tbo-IT2 toxin remains unknown.

In addition to their neurotoxin properties, spider toxins have been found to contain peptidase inhibitors in their digestive systems. These inhibitors serve to deactivate trypsin, a serine peptidase found in their prey. These findings suggest that spiders employ peptidase inhibitors during prey ingestion, adding to the complexity of their venomous strategies [81,82,83,85,86]. Moreover, studies have shown similarities between components in the spider’s digestive system and its venom, including cysteine-rich molecules. Among these, NcTI, identified as a time-dependent inhibitor, has been detected in the digestive juice of the spider *Nephilingis cruentata*, while similar molecules have been observed in other spider species such as *Acanthoscurria geniculata*, *Stegodyphus mimosarum*, and *Loxosceles gaucho* [87,88,89,90,91]. These findings further underscore the intriguing strategies spiders employ in their survival tactics.

Spiders also employ venom as an evolutionary self-defense strategy, inflicting pain on potential threats or attackers [92,93,94,95,96,97,98]. For instance, injecting δ-HXTX-Ar1a into the paws of mice triggered an aggressive response and pain [79]. This venom disrupts the inactivation of Nav channels, crucial in nociceptive signaling, which underlies pain perception. Both δ-HXTX-Ar1a and δ-HXTX Mg1a from the Japanese funnel-web spider exhibit sensitivity to the Nav1.1 and Nav1.6 channels, significant players in pain signaling [92,93,98]. Nav1.6, in particular, is the main subtype of Ranvier node in motor neurons. Consequently, the inhibition of Nav channels by venom peptides, such as δ/κ-TRTX-Pm1a from Pelinobius muticus, a burrowing African tarantula, can potentially induce sensory and motor effects in affected individuals [99]. δ-HXTXs, thus, act as potent tools to deter predators, achieving this by inducing hyperexcitability, enhancing anti-tetrodotoxin sodium currents, impairing repolarization, and lowering action potential discharge thresholds, ultimately leading to severe pain [3,99].

Another study highlighted the intriguing effects of LaFr26, a pore-forming peptide found in Lachesana spider venom. It induced depolarization in 56–3 cells and stimulated burst firing in hippocampal neurons independently of synaptic input. Injection of this peptide into mice also induced hyperalgesic behavior through the spider’s claws [100]. Apart from activating ion channels to trigger pain responses, spider venom can induce pain through other means. Studies have indicated that individuals experiencing *Phoneutria nigriventer* venom intoxication primarily exhibit severe localized pain [101]. This pain can be influenced by various factors, including the activation of tachykinin and glutamate receptors peripherally, as well as the involvement of excitatory amino acids, pro-inflammatory cytokines, nitrogen, and prostaglandins [101]. These findings strongly imply that spider toxins possess the capacity to elicit pain in their prey as a defense mechanism, potentially involving ion channels or other transmission mechanisms.

In nature, species frequently engage in competitive relationships that extend beyond superficial conflicts. Atracotoxins, which are recently identified peptide toxins in the venom of the Australian funnel-web spider, strongly compete with the classical scorpion κ toxin AaH II for the neurotoxin receptor 3 site on the rat brain sodium channel. This competition leads to a slowdown in the inactivation of sodium currents, resembling the effect of scorpion α-toxin [102]. Moreover, nanomolar concentrations of δ-atracotoxin-Hv1 and δ-atracotoxin-Ar1 effectively inhibit the binding of LqhαIT, a highly active scorpion α-toxin against insects, to neuronal membranes in cockroaches [102]. Additionally, δ-atracotoxin-Hv1 synergistically enhances batrachotoxin binding to rat brain synaptosomes, similar to the action of scorpion α-toxin. These findings suggest that δ-atracotoxins can bind to both mammalian and insect sodium channels at sites that are similar to or partially overlapping with the receptor binding sites of scorpion α-toxins [102]. Additionally, studies have demonstrated that low concentrations of δ-atracotoxins completely inhibit the binding of scorpion α-toxin 125I-Lqh II (where Lqh represents α-toxin II from the venom of the scorpion *Leiurus quinquestriatus hebraeus*). This inhibition occurs because these toxins have receptor sites that are similar to or partially overlap with each other [103]. Furthermore, δ-atracotoxins (δ-ACTXs) found in Australian funnel-web spiders have a distinct structure compared to scorpion α-toxins (ScαTx) [104]. However, as with ScαTx, δ-atracotoxins still slow down sodium current inactivation and compete with them for binding to receptor sites 3 on sodium channels. The binding of 125I-labeled δ-ACTX-Hv1a to rat brain synaptosomes was competitively inhibited by typical ScαTx and exhibited a high affinity (Kd = 0.42 nm) for cockroach Na^+^ channels [104]. These findings suggest that spider toxins have competitive binding sites with toxins from other species, which may contribute to their survival advantage in nature.

Spider-venom secretion is a survival strategy for predation, defense, and deterring rivals. Spiders secrete toxins to prey on various insects, such as cockroaches, flies, and crickets, and inflict pain on prey or attackers as an evolutionary self-defense strategy. This pain can be influenced by various factors, including activation of tachykinin and glutamate receptors peripherally, as well as the involvement of excitatory amino acids, pro-inflammatory cytokines, nitrogen, and prostaglandins. In addition, species frequently engage in competitive relationships that extend beyond superficial conflicts. For example, as with ScαTx, δ-ACTXs still slow down sodium current inactivation and compete with them for binding to receptor sites 3 on sodium channels.

## 5. Enabling Research: Spider-Venom Peptide Applications in the Research of Ion Channel and Pharmacological Mechanisms

**Figure 6:**
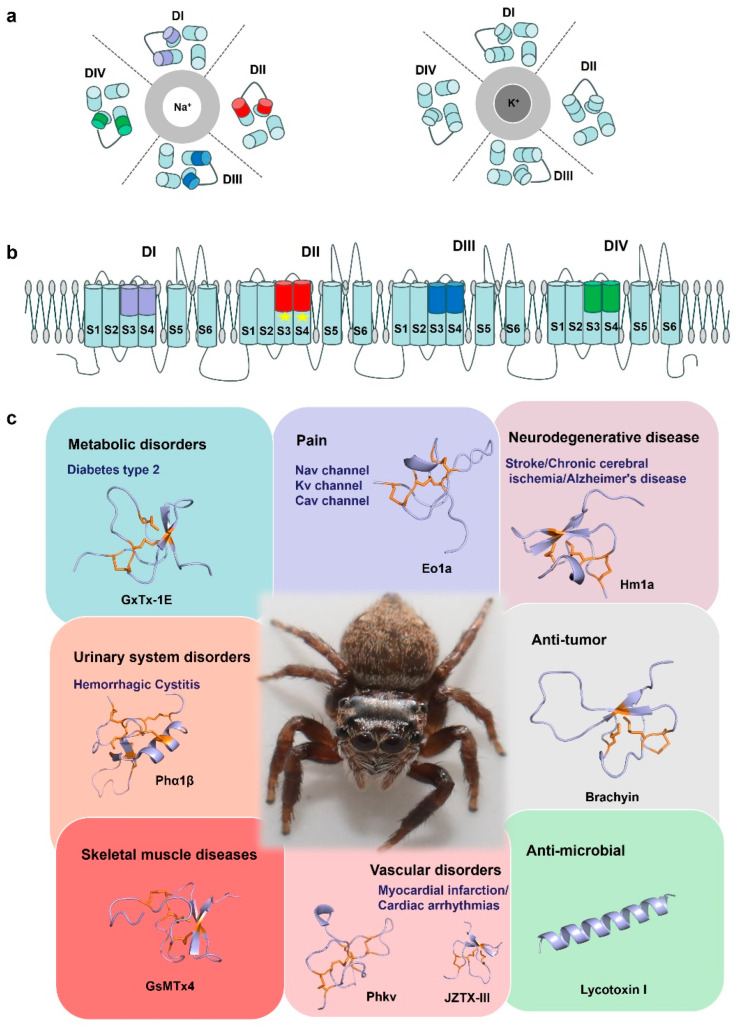
Spider-venom research tools. (**a**) A cartoon of the structure of the Nav channel and Kv channel. The central Na^+^ and K^+^ selective pore is surrounded by the four domains (DI–DIV). (**b**) Cartoon representing a side view of the Nav channel embedded in a lipid membrane. S1–S4: voltage sensor, S5–S6: pore. In general, spider toxin peptides act by focusing on different voltage sensors of a domain on the Nav channels, such as Eo1a binding to the domain II S3–S4 extracellular loop of Nav1.8 to enhance activation. Due to its structural specificity and diverse electrophysiological functions, spider venom is considered a potential tool for elucidating the basic structure-function relationship of ion channels. (**c**) By acting on different targets, spider toxins are of great significance for drug development and clinical treatment of diseases such as chronic pain, neurological diseases, cardiovascular diseases, and have antibacterial and anti-tumor properties. GxTx-1E (UniProt: P84835), Eola (UniProt: P0DW95), Hmla (UniProt: P60992), Phα1β (UniProt: P81792), Brachyin (UniProt: P0DUC0), GsMTx4 (UniProt: P84835), Phkv (UniProt: O76200), JZTX-III (UniProt: P62520), Lycotoxin I (UniProt: P61507). Disulfide bridges are shown in orange.

Spider venoms contain a variety of highly selective ion channel modulators, which are valuable for ion channel research and drug development (Figure 6). Some of the channels have been validated as potential therapeutic targets for neuropathic and chronic pain, irritable bowel syndrome, cardiovascular disease, and epilepsy [92,105,106]. One prominent modulator is the voltage-gated sodium channel Nav1.8, predominantly found in peripheral nociceptive neurons and playing a crucial role in pain sensitization, leading to inflammation and neuropathic pain. Consequently, Nav1.8 has become a promising target for developing analgesics to alleviate pain [107]. The specific spider toxin called µ-TRTX-Hl1a, derived from the *Haplopelma lividum* spider, has been discovered to selectively reduce the current amplitude of Nav1.8 with an IC_50_ of 2.19 µM, without affecting other sodium isoforms [5]. Importantly, µ-TRTX-Hl1a does not affect potassium and calcium channels [5]. Thus, µ-TRTX-Hl1a exhibits remarkable selectivity towards Nav1.8, rendering it a valuable tool for investigating the structure and function of this channel. In another study, the effect of Eo1a, a peptide derived from the venom of the *Tanzanian black and olive baboon tarantula*, on Nav1.8 peak currents was investigated [108]. The study found that Eo1a rapidly inactivated in voltage-dependent activation and steady state, displaying greater selectivity for Nav1.8 in comparison with Nav1.7 [108]. Furthermore, the study revealed that the insertion of the Nav1.8 DII S3-S4 extracellular loop into Nav1.7 yielded analogous results to Nav1.8, indicating that the primary binding site for Eo1a was the Nav1.8 DII S3-S4 extracellular loop [108]. Moreover, the substitution of negatively charged D24 on Eo1a with Lys led to the abolition of Nav1.8 activity [108]. These findings contribute to the development of spider-venom peptides that possess enhanced potency and selectivity against Nav1.8, thus laying the groundwork for future rational design.

Due to their conserved structural scaffolds and diverse electrophysiological functions, spider-venom peptides are considered potential tools for elucidating the fundamental structure-function relationship of voltage-gated sodium channels. For instance, Jingzhaotoxin-III (JZTX-III), a unique sodium channel gating modulator from the *Tarantula Chilobrachys jingzhao* selectively inhibits the activation of cardiac sodium channels, setting it apart from neuronal subtypes [109]. This study’s researchers revealed that the toxin’s binding is critically influenced by two acidic residues (Asp1, Glu3) formed by four Trp residues (residues 8, 9, 28, and 30), as well as exposed hydrophobic regions [109]. Moreover, the restoration of the mutant D816R in Nav1.7 markedly enhanced the sensitivity of neuronal subtypes to JZTX-III [109]. Conversely, the mutation R800D in Nav1.5 reduced the IC_50_ of JZTX-III. These findings suggest that JZTX-III interacts with Nav1.5 through a unique mechanism, in which R800 plays a key role in trapping domain II S3-S4 [109]. In conclusion, JZTX-III represents a novel tool for investigating sodium channel voltage sensors and holds the potential to elucidate the relationships between structure and function, as well as the interactions of toxins with channels. Nav channels are essential in generating and propagating nerve impulses and the most widely targeted ion channels by toxins from venomous organisms. Hm1a, found in the *Togo starburst tarantula*, has been characterized for its ability to selectively activate voltage-gated sodium channels, in particular the Nav1.1 subtype [92]. Heterozygous loss-of-function mutations in the Nav1.1 gene (SCN1A) are responsible for the development of the vast majority of Dravet syndrome cases, a severe early onset pediatric epilepsy coincident with cognitive impairment, deterioration of motor skills, and ataxia [110,111]. Hm1a delayed the inactivation of Nav1.1 and effectively increased the channel activation to improve inhibitory interneuron function [112]. Because of this, Hm1a was considered a promising therapeutic candidate. Hence, these peptides have the potential to be valuable molecular tools in investigating the relationship between the structure and function of Nav channels.

Many spider-venom peptides have a specific affinity for target potassium (K^+^) channels. Known as gating modulator toxins, they attach to the voltage sensor domain of Kv channels, thereby influencing the channels’ voltage-dependent conformational shifts. In a recent study, κ-LhTx-1, a peptide toxin derived from the venom of the *Pandercetes* spider, was identified as a gate-modifying toxin that hinders the activation of the voltage sensor in the Kv4 channel [113]. Notably, κ-LhTx-1 exhibits distinct modulation of Kv4 channel gating, resulting in a stronger voltage-dependent inhibition on Kv4.2/4.3 in comparison with Kv4.1. By exchanging the non-conserved S3b segment between Kv4.1 (_280_FVPK_283_) and Kv4.3 (_275_VMTN_278_) in an experiment, the voltage-dependent phenotype of κ-LhTx-1 inhibition exhibited complete reversal [113]. This reversal emphasized the significance of specific residues, including P282 in Kv4.1, D281 in Kv4.2, and N278 in Kv4.3 [113]. Moreover, the final two residues of each Kv4 channel (P282/K283 in Kv4.1, T280/D281 in Kv4.2, and T277/N278 in KV4.3) may synergistically contribute to the toxin’s action [113]. Furthermore, κ-LhTx-1 demonstrates high affinity, selectivity, and a unique mode of action when interacting with Kv4.2/4.3 channels. This provides valuable insights into the structural characteristics of Kv4 channels. Analogously, Jingzhaotoxin-XII (JZTX-XII) specifically targets the Kv4.1 channel with an IC_50_ value of 0.363 μM, isolated from the venom of the *Chinese Jingzhao tarantula* [114]. Despite sharing 80% sequence identity with phrixotoxin1 (known for its potent inhibition of the Kv4.2 and Kv4.3 channels), JZTX-XII exhibits distinct pharmacological properties. Notably, the substitution of basic residues (Arg22 and Lys27) in phrixotoxin1 with Glu22 and Tyr27 in JZTX-XII alters the surface potential, resulting in a reduced affinity of JZTX-XII for Kv4.2 channels [114]. Structural analysis indicates differences in the charge distribution of the interacting surface, which could potentially impact the specific pharmacology of these toxins. In addition, Jingzhaotoxin-I, -III, and -V (JZTX-I, -III, and -V) are 29–36 amino acid peptides isolated from the venom of Chinese tarantula [115]. Among these, JZTX-III selectively inhibits Kv2.1 channels, JZTX-V exhibits a higher affinity for Kv4.2 channels than Kv2.1 channels, and JZTX-I inhibits Kv2.1 and Kv4.1 channels with lower affinity [115]. Structure-function analysis suggests that toxin affinity is influenced by electrostatic interactions, and the distinct electrostatic anisotropy may be responsible for the varying affinities of the toxins towards the Kv2.1 and Kv4.1 channels. Consequently, JZTXs represent valuable tools for investigating the Kv2 and Kv4 channels [115]. Another study focused on the isolation of TLTx1 from the giant bird-eating tarantula *Theraphosa leblondi* and its effect on Kv4.2 channel activity [116]. This study observed that TLTx1 significantly enhanced the inactivation of Kv4.2, but the effect was observed only in the absence of the proximal N-terminus [116]. This finding suggests that the proximal Kv4.2 N-terminus not only functions as an inactivation domain, but also plays a role in enhancing inactivation activity. Additionally, the study found that TLTx1 sensitivity can be transferred to Kv2.1 channels by replacing the S3-S4 junction region with the corresponding domain from Kv4.2 [116]. Overall, TLTx1 has the potential to act as a gating modifier for Kv4.2 channels. As is well known, Kv channels play their physiological roles in disease and peptide modulators. The *Phoneutria nigriventer* peptide PhKv has shown a remarkable ability to rescue long-term memory in a mouse model of Alzheimer’s disease, due to its selective modulation of A-type transient outward K+ currents [117]. In addition, PhKv also showed an antinociceptive effect in the mouse model of chronic constriction injury [118] and a reduction in aberrant cardiac activity of arrhythmic rat hearts [119]. Consequently, these peptide toxins serve as valuable molecular tools for exploring the structure-function relationship of Kv channels and show potential for the development of novel therapeutics for Kv channel-related diseases [120].

Spider venom also contains modulators of calcium channels (Cav), in addition to sodium and potassium channels. Selective modulation of Cav channel subtypes plays a crucial role in investigating the function of specific Cav channel subtypes in physiological and pathophysiological processes, especially in neurological, neuropsychiatric, and chronic pain disorders [121,122]. Spider venoms contain peptide modulators for ion channels, many of which have unique isoform selectivity, making them a valuable resource of pharmacological tools and potential drug candidates. Phα1β, isolated from the spider *Phoneutria nigriventer,* shows analgesic potential. It selectively inhibits voltage-gated calcium channels, particularly Cav2 channels [123] and transient receptor potential ankyrin 1 TRPA1 channels [124]. Compared to contemporary drugs morphine and Ziconitide (ω-conotoxin MVIIA delivered through an intrathecal pump), Phα1β is more effective, with fewer side effects and is even able to control cancer-related pain in morphine-tolerant mice [125]. During the investigation of subtype-selective blockers of Cav channels, researchers identified a novel 39-residue peptide known as ω-TRTX-Cc1a (Cc1a) [126]. Cc1a shares 67% sequence similarity to the spider toxin ω-TRTX-Hg1a. Cc1a selectively inhibits Cav channels produced by L-type (Cav1.2 and Cav1.3) with IC_50_ values of 825 nM and 2.24 μM, respectively [126]. Experiments using Cc1a peptides modified at the N-terminus or C-terminus demonstrate the significance of the N- and C-termini in voltage-gated ion channel modulation. Cc1a exhibits remarkable subtype selectivity in Cav channels compared to ω-TRTX-Hg1a, indicating its potential as a valuable tool for unraveling the molecular mechanisms responsible for subtype selectivity in voltage-gated ion channels [126]. Therefore, gating modulator toxins are valuable tools for investigating the state transitions associated with structural changes during channel gating.

## 6. Concluding Remarks

Spider-venom peptides have drawn significant attention from pharmaceutical research due to their great potential in medicine and biotechnology. However, the application of spider-venom peptides in pharmaceuticals faces many challenges and limitations that researchers have been striving to overcome.

A primary challenge stems from the intricate and diverse nature of spider venom. The vast number of spider species and their unique venom compositions make it challenging to comprehensively study the components of venom peptides. Additionally, due to its small size and minimal venom secretion, obtaining sufficient quantities of venom for detailed analysis, such as structure identification, bioactivity evaluation, and research of mechanism, using only conventional chemical and biological techniques, is extremely challenging. Furthermore, some spider-venom peptides may be subject to rapid proteolysis, which limits the route of administration and the effect of drug therapy. Therefore, to date, only very few spider-venom peptides have been used in drug therapy.

Recent technological and methodological advancements such as high-throughput screening techniques, transcriptomics, and proteomics, have played a pivotal role in overcoming these constraints. Synthetic biology and peptide synthesis breakthroughs have also facilitated the chemical synthesis and modification of spider-venom peptides. Based on these new techniques, researchers have improved proteolytic tolerance by C-terminal amidation, N-terminal pyroglutamate protection, peptides cyclization, substitution of D-amino acids.

Looking forward, the future of spider-venom peptide research holds exciting prospects. The potential therapeutic applications of spider-venom peptides, especially in areas such as pain management and antimicrobial drug development, present a promising direction for future research. Further exploration of the untapped diversity of spider venoms, combined with advanced bioinformatics, will likely reveal novel peptides with unique functionalities.

## Figures and Tables

**Table 1:** Antimicrobial peptides/proteins of spider venom.

Toxin	Source	Sequence	References
Juruin	*Avicularia juruensis*	FTCAISCDIKVNGKPCKGSGEKKCSGGWSCKFNVCVKV-OH ^a^	[36]
Lycotoxin I	*Lycosa carolinensis*	IWLTALKFLGKHAAKHLAKQQLSKL-NH_2_	[24]
Lycotoxin II	*Lycosa carolinensis*	KIKWFKTMKSIAKFIAKEQMKKHLGGE-OH	[24]
Lycocitin 1	*Lycosa singoriensis*	GKLQAFLAKMKEIAAQTL-NH_2_	[37]
Lycocitin 2	*Lycosa singoriensis*	GRLQAFLAKMKEIAAQTL-NH_2_	[37]
Ltc 1	*Lachesana tarabaevi*	SMWSGMWRRKLKKLRNALKKKLKGE-OH	[38]
Ltc 2a	*Lachesana tarabaevi*	GLFGKLIKKFGRKAISYAVKKARGKH-OH	[38]
Ltc 3a	*Lachesana tarabaevi*	SWKSMAKKLKEYMEKLKQRA-NH_2_	[38]
Ltc 3b	*Lachesana tarabaevi*	SWASMAKKLKEYMEKLKQRA-NH_2_	[38]
Ltc 4a	*Lachesana tarabaevi*	GLKDKFKSMGEKLKQYIQTWKAKF-NH_2_	[38]
Ltc 4b	*Lachesana tarabaevi*	SLKDKVKSMGEKLKQYIQTWKAKF-NH_2_	[38]
Ltc 5	*Lachesana tarabaevi*	GFFGKMKEYFKKFGASFKRRFANLKKRL-NH_2_	[38]
CIT 1a	*Lachesana tarabaevi*	GFFGNTWKKIKGKADKIMLKKAVKIMVKKEGISKEEAQAKVDAMSKKQIRLYLLKYYGKKALQKASEKL-OH	[38]
LyeTx I	*Lycosa erythrognatha*	IWLTALKFLGKNLGKHLAKQQLAKL-OH	[39]
Cupiennin 1a	*Cupiennius salei*	GFGALFKFLAKKVAKTVAKQAAKQGAKYVVNKQME-NH_2_	[40]
LyeTx I	*Lycosa erythrognatha*	IWLTALKFLGKNLGKHLAKQQLAKL-NH_2_	[41]
U1-SCRTX-Lg1a	*Loxosceles gaucho*	VGTDFSGNDDISDVQK-NH_2_	[42]
Lycotoxin-Pa4a	*Pardosa astrigera*	AMMAESRKDNCIPKHHECTSRPKDCCKQNLMQFKCSCMTIIDKNNKETERCKCDNSIFQKVAKTSVNIGKAVVTK-OH ^b^	[43]
U5-Lycotoxin-Ls1a	*Lycosa tarantula*	FSLARKDKENCIPKHHECTSDRHGCCRGSMFKYKCQCVKIVNAQKEETERCACITPGLHKAAEFVVQLFKKVIA-OH ^c^	[43]
Lyp2370	*Lycosa poonaensis*	FLASHVAMEQLSKLGSKIATKL-NH_2_	[44]
Lyp1987	*Lycosa poonaensis*	G/RLQAFLAKMKEIAAQTL-NH_2_	[44]
U1-TRTX-Ar1a	*Acanthoscurria* *rondoniae*	SCVHERETCSKVRGPLCCRGECTCPIYGDCFCYGS-OH ^c^	[45]

The sequence of spider-venom peptides containing disulfide bonds have been labeled and the cysteines are colored red. ^a^ The sequence contains three pairs of disulfide bridges (C1–C4, C2–C5, C3–C6). ^b^ The sequence contains four pairs of disulfide bridges (C1–C4, C2–C5, C3–C8, C6–C7). ^c^ The sequence contains four pairs of disulfide bridges (C1–C4, C2–C5, C3–C8, C6–C7). The sequence predicted disulfide bond connectivities that have not been experimentally validated.

**Table 2:** Anticancer peptides/proteins of spider venom.

Toxin	Source	Sequence	References
Brachyin	*Brachypelma albopilosum*.	CLGENVPCDKDRPNCCSRYECLEPTGYGWWYASYYCYKKRS-OH ^a^	[21]
Lycosin-I	*Lycosa singorensis*	RKGWFKAMKSIAKFIAKEKLKEHL-OH	[24]
*Gomesin*	*Acanthoscurria gomesiana*	GCRRLCYKQRCVTYCRGR-OH ^b^	[53]
PcTx-1	*Psalmopoeus cambridgei*	EDCIPKWKGCVNRHGDCCEGLECWKRRRSFEVCVPKTPKT-OH ^a^	[54]
Latarcin 2a	*Lachesana tarabaevi*	GLFGKLIKKFGRKAISYAVKKARGKH-OH	[55]
U1-TRTX-Agm3a	*Acanthoscurria rondoniae*	ACGSFMWKCSERLPCCQEYVCSPQWKWCQNP-OH ^a^	[45]
U1-TRTX-Ar1b	*Acanthoscurria rondoniae*	SCVYERETCSKVRGPLCCRGECTCPIYGDCFCYGS-OH ^c^	[45]
SNX-482	*Hysterocrates gigas*	GVDKAGCRYMFGGCSVNDDCCPRLGCHSLFSYCAWDLTFSD-OH ^a^	[56]
LVTX-9	*Lycosa vittata*	ASIGALIQKAIALIKAKAA-NH_2_	[57]
LVTX-8	*Lycosa vittata*	IWLTALKFLGKNLGKHLAKQQLSKL-NH_2_	[57]

The sequence of spider-venom peptides containing disulfide bonds have been labeled and the cysteines are colored red. ^a^ The sequence contains three pairs of disulfide bridges (C1–C4, C2–C5, C3–C6). ^b^ The sequence contains two pairs of disulfide bridges (C1–C4, C2–C3). ^c^ The sequence contains four pairs of disulfide bridges (C1–C4, C2–C5, C3–C8, C6–C7). The sequence predicted disulfide bond connectivities that have not been experimentally validated.

**Table 3:** Insecticidal spider peptides/proteins (ISPs).

Toxin Name	Source	Sequence	References
β-diguetoxin-Dc1a	*Diguetia canities*	SAKDGDVEGPAGCKKYDVECDSGECCQKQYLWYKWRPLDCRCLKSGFFSSKCVCRDV-OH ^a^	[58]
CsTx-1	*Cupiennius salei*	SCIPKHEECTNDKHNCCRKGLFKLRCQCSTFDDESGQPTEFCACGRPMGHQAIETGLNIFRGLFKGKKKNKKTK-OH ^a^	[60]
CsTx-2a	*Cupiennius salei*	SCIPKHEECTNDKHNCCRKGLFKLCQCSTFDDESGQPTERCACGRPMGHQAIETGLNIFRGLFA-OH ^a^	[60]
CsTx-2b	*Cupiennius salei*	SCIPKHEECTNDKHNCCRKGLFKLCQCSTFDDESGQPTERCACGRPMGHQAIETGLNIFRGLF-OH ^a^	[60]
CsTx-13	*Cupiennius salei*	SDCTLRNNDCTDDRHSCCRSKMFKDVCTCFYPSQAKKELCTCQQPKHLKYIEKGLQKAKDYAT-OH ^a^	[16]
Osu1	*Oculicosa supermirabilis*	RLALPPGAVCNGHKSDCQCFGAKYKCSCPFFWRFRKSAECHCKKGWAWTAIKKRSCHNRYQWSD-OH ^b^	[61]
ω-hexatoxin-Hvn1b	*Hadronyche venenata*	SPTCIPSGQPCPYNENCCSKSCTYKENENGNTVQRCD-OH ^c^	[62]
JFTX-23	*Selenocosmia jiafu*	QRACGQLHDPCPNDRPGHRTCCLGLQCRYGNCLVQV-OH ^c^	[63]
β/δ-PrIT1	*Phoneutria reidyi*	CGDINAPCQSDCDCCGYSVTCDCYWGNECKCRESNFAIGM-OH ^a^	[64]
CpTx1	*Cheiracanthium auditorium*	GKTCIERNKECTNDRHGCCRGKIFKDKCTCVKNGKTEKCVCTQKKWAKIIESYIGDIPALPKPV-NH_2_^a^	[65]
CpTx2a	*Cheiracanthium punctorium*	GKKCIERNKECTNDRHGCCRGKIFKDKCECVGSGGKERRCVCKQKKWAKIIESYIGDIPTLPKPE-NH_2_^a^	[66]
CpTx3a	*Cheiracanthium punctorium*	TCVPRDGDCTENRKACCRSKIFQDRCQCRKVSQDKVACSCKQPYWLMKIEEILGDIPEKPKPV-NH_2_^a^	[66]
CpTx4a	*Cheiracanthium punctorium*	ASCTERKHDCTKDRHSCCRGKIFKDKCTCVKNGKTEKCVCTQKKWAKIIESYIGDIPALPKPV-NH_2_^a^	[66]
Magi3	*Macrothele gigas*	GGCIKWNHSCQTTTLKCCGKCVVCYCHTPWGTNCRCDRTRLFCTED-OH ^d^	[67]
OxyTx1	*Oxyopes lineatus*	DWECLPLHSSCDNDCVCCKNHHCHCPYSNVSKLEKWLPEWAKIPDALKRCSCQRNDKDGKINTCDKYKN-NH_2_^e^	[68]
OxyTx2	*Oxyopes lineatus*	AWKCLPKDSTCGDDCDCCEGLHCHCPLRNMLPAILRCSCQSKDDHINTCPKYKKS-NH_2_^e^	[68]
OtTx1a	*Oxyopes takobius*	GTPVGNNKCWAIGTTCSDDCDCCPEHHCHCPAGKWLPGLFRCTCOVTESDKVVNKCPPAE-OH ^e^	[69]
ω-Tbo-IT1	*Tibellus oblongus*	CASKNERCGNALYGTKGPGCCNGKCICRTVPRKGVNSCRCM-OH ^a^	[70]
U2-SCTX-Li1b	*Loxosceles intermedia*	GCIKSGQRCGSPHGLPSNCCDDWKYKGRCGCTMGVCTCGKNCPSRGCDYRTKG-OH ^a^	[71]
μ-SPRTX-Hv2	*Heteropoda venatoria*	DDDCGKLFADCTSDSDCCENWVCSKTGFVKNICKYNF-OH ^c^	[72]
μ-TRTX-Ae1a	*Augacephalus ezendami*	GVDKEGCRYLLGACTIDDDCCLHLGCNKKYGHCGWDTF-OH ^c^	[59]
brachyin	*Brachypelma albopilosum*	CLGENVPCDKDRPNCCSRYECLEPTGYGWWYASYYCYKKRS-OH ^c^	[21]
β-TRTX-Cd1a	*Ceratogyrus darlingi*	DCLGWFKSCDPKNDKCCKNYSCSRRDRWCKYDL-OH ^c^	[73]
U1-TRTX-Ct1a	*Coremiocnemis tropix*	LFECSFSCDIKKNGKPCKGSGEKKCSGGWRCKMNFCVKV-OH ^f^	[74]
U1-TRTX-Ct1b	*Coremiocnemis tropix*	FECSLSCDIKKNGKPCKGSGEKKCSGGWRCKMNFCLKF-OH ^f^	[74]
μ/ω-TRTX-Mb1a	*Monocentropus balfouri*	GVDKPGCRYMFGGCVQDDDCCPHLGCKRKGLYCAWDGT-OH ^c^	[75]
μ/ω-TRTX-Mb1b	*Monocentropus balfouri*	GVDKPGCRYMFGGCVQDDDCCPHLGCKRKGLYCAWDAS-OH ^c^	[75]
OAIP-1	*Selenotypus plumipes*	DCGHLHDPCPNDRPGHRTCCIGLQCRYGKCLVRV-NH_2_^c^	[76]
Latroeggtoxin-III	*Latrodectus tredecimguttatus*	STKSSESLYLEALYIDKMTHEPVAD (N-terminal sequence)	[77]

The sequence of spider-venom peptides containing disulfide bonds have been labeled and the cysteines are colored red. ^a^ The sequence contains four pairs of disulfide bridges (C1–C4, C2–C5, C3–C8, C6–C7). ^b^ The sequence contains four pairs of disulfide bridges (C1–C4, C2–C8, C3–C7, C5–C6). The sequence predicted disulfide bond connectivities that have not been experimentally validated. ^c^ The sequence contains three pairs of disulfide bridges (C1–C4, C2–C5, C3–C6). ^d^ The sequence contains five pairs of disulfide bridges (C1–C4, C2–C6, C3–C9, C5–C10, C7–C8). ^e^ The sequence contains five pairs of disulfide bridges (C1–C5, C2–C6, C3–C10, C4–C9, C7–C8). ^f^ The sequence contains three pairs of disulfide bridges (C1–C3, C2–C5, C4–C6).

## Data Availability

No new data were created or analyzed in this study. Data sharing is not applicable to this article.

## References

[1] Vetter R.S., Isbister G.K. (2008). Medical aspects of spider bites. Annu. Rev. Entomol..

[2] Hauke T.J., Herzig V. (2017). Dangerous arachnids—Fake news or reality?. Toxicon.

[3] Shaikh N.Y., Sunagar K. (2023). The deep-rooted origin of disulfide-rich spider venom toxins. eLife.

[4] Li P., Zhang Z., Liao Q., Meng E., Mwangi J., Lai R., Rong M. (2020). LCTX-F2, a Novel Potentiator of Coagulation Factors from the Spider Venom of Lycosa singoriensis. Front. Pharmacol..

[5] Meng P., Huang H., Wang G., Yang S., Lu Q., Liu J., Lai R., Rong M. (2016). A Novel Toxin from Haplopelma lividum Selectively Inhibits the Na(V)1.8 Channel and Possesses Potent Analgesic Efficacy. Toxins.

[6] Rash L.D., Hodgson W.C. (2002). Pharmacology and biochemistry of spider venoms. Toxicon.

[7] Saez N.J., Herzig V. (2019). Versatile spider venom peptides and their medical and agricultural applications. Toxicon.

[8] Escoubas P., Sollod B., King G.F. (2006). Venom landscapes: Mining the complexity of spider venoms via a combined cDNA and mass spectrometric approach. Toxicon.

[9] Saez N.J., Senff S., Jensen J.E., Er S.Y., Herzig V., Rash L.D., King G.F. (2010). Spider-venom peptides as therapeutics. Toxins.

[10] Vassilevski A.A., Kozlov S.A., Grishin E.V. (2009). Molecular diversity of spider venom. Biochemistry.

[11] King G.F., Hardy M.C. (2013). Spider-venom peptides: Structure; pharmacology, and potential for control of insect pests. Annu. Rev. Entomol..

[12] Yuan F.C., Sun F.D., Zhang L., Huang B., An H.L., Rong M.Q., Du C.W. (2022). General mechanism of spider toxin family I acting on sodium channel Nav1.7. Zool. Res..

[13] Jian-Yi P.A.N., Wei-Jun H.U., Song-Ping L. (2002). Purification, Sequencing and Characterization of Hainantoxin-VI, a Neuro Toxin from the Chinese Bird Spider *Selenocosmia hainana*. Zool. Res..

[14] Wang Z., Chen J., Babicheva A., Jain P.P., Rodriguez M., Ayon R.J., Ravellette K.S., Wu L., Balistrieri F., Tang H. (2021). Endothelial upregulation of mechanosensitive channel Piezo1 in pulmonary hypertension. Am. J. Physiol.-Cell Physiol..

[15] Diniz M.R.V., Paiva A.L.B., Guerra-Duarte C., Nishiyama M.Y., Mudadu M.A., Oliveira U., Borges M.H., Yates J.R., Junqueira-de-Azevedo I.L. (2018). An overview of Phoneutria nigriventer spider venom using combined transcriptomic and proteomic approaches. PLoS ONE.

[16] Clemencon B., Kuhn-Nentwig L., Langenegger N., Kopp L., Peigneur S., Tytgat J., Nentwig W., Lüscher B.P. (2020). Neurotoxin Merging: A Strategy Deployed by the Venom of the Spider *Cupiennius salei* to Potentiate Toxicity on Insects. Toxins.

[17] Craik D.J., Daly N.L., Waine C. (2001). The cystine knot motif in toxins and implications for drug design. Toxicon.

[18] Shekh S., Moi S., Govindu P.C.V., Gowd K.H. (2022). Conformations of disulfides are conserved in inhibitory cystine knot (ICK) motif polypeptides. Toxicon.

[19] Wang X., Connor M., Smith R., Maciejewski M.W., Howden M.E., Nicholson G.M., Christie M.J., King G.F. (2000). Discovery and characterization of a family of insecticidal neurotoxins with a rare vicinal disulfide bridge. Nat. Struct. Biol..

[20] Mourão C.B., Heghinian M.D., Barbosa E.A., Marí F., Bloch C., Restano-Cassulini R., Possani L.D., Schwartz E.F. (2013). Characterization of a novel peptide toxin from *Acanthoscurria paulensis* spider venom: A distinct cysteine assignment to the HWTX-II family. Biochemistry.

[21] Zhong Y., Song B., Mo G., Yuan M., Li H., Wang P., Yuan M., Lu Q. (2014). A novel neurotoxin from venom of the spider, Brachypelma albopilosum. PLoS ONE.

[22] Langenegger N., Nentwig W., Kuhn-Nentwig L. (2019). Spider Venom: Components, Modes of Action, and Novel Strategies in Transcriptomic and Proteomic Analyses. Toxins.

[23] Akef H.M. (2018). Anticancer; antimicrobial, and analgesic activities of spider venoms. Toxicol. Res..

[24] Liu Z., Deng M., Xiang J., Ma H., Hu W., Zhao Y., Li D.W., Liang S. (2012). A novel spider peptide toxin suppresses tumor growth through dual signaling pathways. Curr. Mol. Med..

[25] Hughes S.R., Dowd P.F., Johnson E.T. (2012). Cell-penetrating recombinant peptides for potential use in agricultural pest control applications. Pharmaceuticals.

[26] Garcia F., Villegas E., Espino-Solis G.P., Rodriguez A., Paniagua-Solis J.F., Sandoval-Lopez G., Possani L.D., Corzo G. (2013). Antimicrobial peptides from arachnid venoms and their microbicidal activity in the presence of commercial antibiotics. J. Antibiot..

[27] Megaly A.M.A., Miyashita M., Abdel-Wahab M., Nakagawa Y., Miyagawa H. (2022). Molecular Diversity of Linear Peptides Revealed by Transcriptomic Analysis of the Venom Gland of the Spider *Lycosa poonaensis*. Toxins.

[28] Wadhwani P., Sekaran S., Strandberg E., Bürck J., Chugh A., Ulrich A.S. (2021). Membrane Interactions of Latarcins: Antimicrobial Peptides from Spider Venom. Int. J. Mol. Sci..

[29] Gaza J.T., Leyson J.J.C., Peña G.T., Nellas R.B. (2021). pH-Dependent Conformations of an Antimicrobial Spider Venom Peptide, Cupiennin 1a, from Unbiased HREMD Simulations. ACS Omega.

[30] Kuznetsov A.S., Dubovskii P.V., Vorontsova O.V., Feofanov A.V., Efremov R.G. (2014). Interaction of linear cationic peptides with phospholipid membranes and polymers of sialic acid. Biochemistry.

[31] Idiong G., Won A., Ruscito A., Leung B.O., Hitchcock A.P., Ianoul A. (2011). Investigating the effect of a single glycine to alanine substitution on interactions of antimicrobial peptide latarcin 2a with a lipid membrane. Eur. Biophys. J..

[32] Li F., Wu S., Chen N., Zhu J., Zhao X., Zhang P., Zeng Y., Liu Z. (2021). Fatty Acid Modification of the Anticancer Peptide LVTX-9 to Enhance Its Cytotoxicity against Malignant Melanoma Cells. Toxins.

[33] Won A., Khan M., Gustin S., Akpawu A., Seebun D., Avis T.J., Leung B.O., Hitchcock A.P., Ianoul A. (2011). Investigating the effects of L- to D-amino acid substitution and deamidation on the activity and membrane interactions of antimicrobial peptide anoplin. Biochim. Biophys. Acta.

[34] Oparin P.B., Nadezhdin K.D., Berkut A.A., Arseniev A.S., Grishin E.V., Vassilevski A.A. (2016). Structure of purotoxin-2 from wolf spider: Modular design and membrane-assisted mode of action in arachnid toxins. Biochem. J..

[35] Kuzmenkov A.I., Fedorova I.M., Vassilevski A.A., Grishin E.V. (2013). Cysteine-rich toxins from Lachesana tarabaevi spider venom with amphiphilic C-terminal segments. Biochim. Biophys. Acta.

[36] Ayroza G., Ferreira I.L., Sayegh R.S., Tashima A.K., Da S.J.P.I. (2012). Juruin: An antifungal peptide from the venom of the Amazonian Pink Toe spider, *Avicularia juruensis*, which contains the inhibitory cystine knot motif. Front. Microbiol..

[37] Budnik B.A., Olsen J.V., Egorov T.A., Anisimova V.E., Galkina T.G., Musolyamov A.K., Grishin E.V., Zubarev R.A. (2004). De novo sequencing of antimicrobial peptides isolated from the venom glands of the wolf spider Lycosa singoriensis. J. Mass Spectrom..

[38] Kozlov S.A., Vassilevski A.A., Feofanov A.V., Surovoy A.Y., Karpunin D.V., Grishin E.V. (2006). Latarcins, antimicrobial and cytolytic peptides from the venom of the spider *Lachesana tarabaevi* (Zodariidae) that exemplify biomolecular diversity. J. Biol. Chem..

[39] Santos D.M., Verly R.M., Piló-Veloso D., de Maria M., de Carvalho M.A., Cisalpino P.S., Soares B.M., Diniz C.G., Farias L.M., Moreira D.F. (2010). LyeTx I, a potent antimicrobial peptide from the venom of the spider *Lycosa erythrognatha*. Amino Acids.

[40] Kuhn-Nentwig L., Sheynis T., Kolusheva S., Nentwig W., Jelinek R. (2013). N-terminal aromatic residues closely impact the cytolytic activity of cupiennin 1a, a major spider venom peptide. Toxicon.

[41] Fuscaldi L.L., de Avelar Júnior J.T., Dos Santos D.M., Boff D., de Oliveira V.L.S., Gomes K.A.G.G., Cruz R.C., de Oliveira P.L., Magalhães P.P., Cisalpino P.S. (2021). Shortened derivatives from native antimicrobial peptide LyeTx I: In vitro and in vivo biological activity assessment. Exp. Biol. Med..

[42] Segura-Ramirez P.J., Silva J.P.I. (2018). Loxosceles gaucho Spider Venom: An Untapped Source of Antimicrobial Agents. Toxins.

[43] Shin M.K., Hwang I.W., Kim Y., Kim S.T., Jang W., Lee S., Bang W.Y., Bae C.H., Sung J.S. (2020). Antibacterial and Anti-Inflammatory Effects of Novel Peptide Toxin from the Spider *Pardosa astrigera*. Antibiotics.

[44] Megaly A.M.A., Yoshimoto Y., Tsunoda Y., Miyashita M., Abdel-Wahab M., Nakagawa Y., Miyagawa H. (2021). Characterization of 2 linear peptides without disulfide bridges from the venom of the spider *Lycosa poonaensis* (Lycosidae). Biosci. Biotechnol. Biochem..

[45] Camara G.A., Nishiyama-Jr M.Y., Kitano E.S., Oliveira U.C., da Silva P.I., Junqueira-de-Azevedo I.L., Tashima A.K. (2020). A Multiomics Approach Unravels New Toxins with Possible In Silico Antimicrobial, Antiviral, and Antitumoral Activities in the Venom of *Acanthoscurria rondoniae*. Front. Pharmacol..

[46] Jemal A., Bray F., Center M.M., Ferlay J., Ward E., Forman D. (2011). Global cancer statistics. CA Cancer J. Clin..

[47] Wang Y., Yan Q., Fan C., Mo Y., Wang Y., Li X., Liao Q., Guo C., Li G., Zeng Z. (2023). Overview and countermeasures of cancer burden in China. Sci. China Life Sci..

[48] Baskar R., Lee K.A., Yeo R., Yeoh K.W. (2012). Cancer and radiation therapy: Current advances and future directions. Int. J. Med. Sci..

[49] Liberio M.S., Joanitti G.A., Fontes W., Castro M.S. (2013). Anticancer peptides and proteins: A panoramic view. Protein Pept. Lett..

[50] Liang H., Lu Q., Yang J., Yu G. (2023). Supramolecular Biomaterials for Cancer Immunotherapy. Research.

[51] Al-Asmari A.K., Riyasdeen A., Al-Shahrani M.H., Islam M. (2016). Snake venom causes apoptosis by increasing the reactive oxygen species in colorectal and breast cancer cell lines. Onco Targets Ther..

[52] Tan H., Luo W., Wei L., Chen B., Li W., Xiao L., Manzhos S., Liu Z., Liang S. (2016). Quantifying the Distribution of the Stoichiometric Composition of Anticancer Peptide Lycosin-I on the Lipid Membrane with Single Molecule Spectroscopy. J. Phys. Chem. B.

[53] Barbosa F.M., Daffre S., Maldonado R.A., Miranda A., Nimrichter L., Rodrigues M.L. (2007). Gomesin, a peptide produced by the spider *Acanthoscurria gomesiana*, is a potent anticryptococcal agent that acts in synergism with fluconazole. FEMS Microbiol. Lett..

[54] Rooj A.K., McNicholas C.M., Bartoszewski R., Bebok Z., Benos D.J., Fuller C.M. (2012). Glioma-specific cation conductance regulates migration and cell cycle progression. J. Biol. Chem..

[55] Dubovskii P.V., Vassilevski A.A., Kozlov S.A., Feofanov A.V., Grishin E.V., Efremov R.G. (2015). Latarcins: Versatile spider venom peptides. Cell Mol. Life Sci..

[56] Munhoz J., Thomé R., Rostami A., Ishikawa L.L.W., Verinaud L., Rapôso C. (2021). The SNX-482 peptide from *Hysterocrates gigas* spider acts as an immunomodulatory molecule activating macrophages. Peptides.

[57] Zhang P., Yan Y., Wang J., Dong X., Zhang G., Zeng Y., Liu Z. (2020). An Anti-Cancer Peptide LVTX-8 Inhibits the Proliferation and Migration of Lung Tumor Cells by Regulating Causal Genes’ Expression in p53-Related Pathways. Toxins.

[58] Bende N.S., Dziemborowicz S., Mobli M., Herzig V., Gilchrist J., Wagner J., Nicholson G.M., King G.F., Bosmans F. (2014). A distinct sodium channel voltage-sensor locus determines insect selectivity of the spider toxin Dc1a. Nat. Commun..

[59] Herzig V., Ikonomopoulou M., Smith J.J., Dziemborowicz S., Gilchrist J., Kuhn-Nentwig L., Rezende F.O., Moreira L.A., Nicholson G.M., Bosmans F. (2016). Molecular basis of the remarkable species selectivity of an insecticidal sodium channel toxin from the African spider *Augacephalus ezendami*. Sci. Rep..

[60] Kuhn-Nentwig L., Fedorova I.M., Lüscher B.P., Kopp L.S., Trachsel C., Schaller J., Vu X.L., Seebeck T., Streitberger K., Nentwig W. (2012). A venom-derived neurotoxin, CsTx-1, from the spider Cupiennius salei exhibits cytolytic activities. J. Biol. Chem..

[61] Alvarado D., Cardoso-Arenas S., Corrales-García L.L., Clement H., Arenas I., Montero-Dominguez P.A., Olamendi-Portugal T., Zamudio F., Csoti A., Borrego J. (2020). A Novel Insecticidal Spider Peptide that Affects the Mammalian Voltage-Gated Ion Channel hKv1.5. Front. Pharmacol..

[62] Wei Z., Xu J., Peng X., Yuan Z., Zhao C., Guo K., Zhang X., He Y., Zhang Z., Wu Y. (2022). Preparation and performance characteristics of spider venom peptide nanocapsules. Pest. Manag. Sci..

[63] Monfared N., Ahadiyat A., Fathipour Y., Mianroodi R.A. (2022). Evaluation of recombinant toxin JFTX-23, an oral-effective anti-insect peptide from the spider Selenocosmia jiafu venom gland proteome. Toxicon.

[64] de Oliveira L.C., Campos F.V., Figueiredo S.G., Cordeiro M.N., Adaime B.R., Richardson M., Pimenta A.M., Martin-Eauclaire M.F., Beirão P.S., De Lima M.E. (2015). β/δ-PrIT1, a highly insecticidal toxin from the venom of the Brazilian spider *Phoneutria reidyi* (F.O. Pickard-Cambridge, 1897). Toxicon.

[65] Vassilevski A.A., Fedorova I.M., Maleeva E.E., Korolkova Y.V., Efimova S.S., Samsonova O.V., Schagina L.V., Feofanov A.V., Magazanik L.G., Grishin E.V. (2010). Novel class of spider toxin: Active principle from the yellow sac spider *Cheiracanthium punctorium* venom is a unique two-domain polypeptide. J. Biol. Chem..

[66] Sachkova M.Y., Slavokhotova A.A., Grishin E.V., Vassilevski A.A. (2014). Structure of the yellow sac spider *Cheiracanthium punctorium* genes provides clues to evolution of insecticidal two-domain knottin toxins. Insect Mol. Biol..

[67] Titaux-Delgado G., Carrillo E., Mendoza A., Mayorga-Flores M., Escobedo-González F.C., Cano-Sánchez P., López-Vera E., Corzo G., Del Rio-Portilla F. (2018). Successful refolding and NMR structure of rMagi3: A disulfide-rich insecticidal spider toxin. Protein Sci..

[68] Estrada G., Silva A.O., Villegas E., Ortiz E., Beirão P.S., Corzo G. (2016). Heterologous expression of five disulfide-bonded insecticidal spider peptides. Toxicon.

[69] Vassilevski A.A., Sachkova M.Y., Ignatova A.A., Kozlov S.A., Feofanov A.V., Grishin E.V. (2013). Spider toxins comprising disulfide-rich and linear amphipathic domains: A new class of molecules identified in the lynx spider *Oxyopes takobius*. FEBS J..

[70] Mikov A.N., Fedorova I.M., Potapieva N.N., Maleeva E.E., Andreev Y.A., Zaitsev A.V., Kim K.K., Bocharov E.V., Bozin T.N., Altukhov D.A. (2015). ω-Tbo-IT1–New Inhibitor of Insect Calcium Channels Isolated from Spider Venom. Sci. Rep..

[71] Matsubara F.H., Meissner G.O., Herzig V., Justa H.C., Dias B.C., Trevisan-Silva D., Gremski L.H., Gremski W., Senff-Ribeiro A., Chaim O.M. (2017). Insecticidal activity of a recombinant knottin peptide from *Loxosceles intermedia* venom and recognition of these peptides as a conserved family in the genus. Insect Mol. Biol..

[72] Xiao Z., Zhang Y., Zeng J., Liang S., Tang C., Liu Z. (2018). Purification and Characterization of a Novel Insecticidal Toxin, mu-sparatoxin-Hv2, from the Venom of the Spider *Heteropoda venatoria*. Toxins.

[73] Sousa S.R., Wingerd J.S., Brust A., Bladen C., Ragnarsson L., Herzig V., Deuis J.R., Dutertre S., Vetter I., Zamponi G.W. (2017). Discovery and mode of action of a novel analgesic β-toxin from the African spider *Ceratogyrus darlingi*. PLoS ONE.

[74] Ikonomopoulou M.P., Smith J.J., Herzig V., Pineda S.S., Dziemborowicz S., Er S.Y., Durek T., Gilchrist J., Alewood P.F., Nicholson G.M. (2016). Isolation of two insecticidal toxins from venom of the Australian theraphosid spider *Coremiocnemis tropix*. Toxicon.

[75] Smith J.J., Herzig V., Ikonomopoulou M.P., Dziemborowicz S., Bosmans F., Nicholson G.M., King G.F. (2017). Insect-Active Toxins with Promiscuous Pharmacology from the African Theraphosid Spider *Monocentropus balfouri*. Toxins.

[76] Hardy M.C., Daly N.L., Mobli M., Morales R.A., King G.F. (2013). Isolation of an orally active insecticidal toxin from the venom of an Australian tarantula. PLoS ONE.

[77] Lei Q., Yu H., Peng X., Yan S., Wang J., Yan Y., Wang X. (2015). Isolation and preliminary characterization of proteinaceous toxins with insecticidal and antibacterial activities from black widow spider (*L. tredecimguttatus*) eggs. Toxins.

[78] Jin L., Fang M., Chen M., Zhou C., Ombati R., Hakim M.A., Mo G., Lai R., Yan X., Wang Y. (2017). An insecticidal toxin from *Nephila clavata* spider venom. Amino Acids.

[79] Herzig V., Sunagar K., Wilson D.T.R., Pineda S.S., Israel M.R., Dutertre S., McFarland B.S., Undheim E.A.B., Hodgson W.C., Alewood P.F. (2020). Australian funnel-web spiders evolved human-lethal δ-hexatoxins for defense against vertebrate predators. Proc. Natl. Acad. Sci. USA.

[80] Korolkova Y., Maleeva E., Mikov A., Lobas A., Solovyeva E., Gorshkov M., Andreev Y., Peigneur S., Tytgat J., Kornilov F. (2021). New insectotoxin from *Tibellus oblongus* spider venom presents novel adaptation of ICK fold. Toxins.

[81] Windley M.J., Herzig V., Dziemborowicz S.A., Hardy M.C., King G.F., Nicholson G.M. (2012). Spider-venom peptides as bioinsecticides. Toxins.

[82] Nicholson G.M. (2007). Insect-selective spider toxins targeting voltage-gated sodium channels. Toxicon.

[83] King G.F., Escoubas P., Nicholson G.M. (2008). Peptide toxins that selectively target insect NaV and CaV channels. Channels.

[84] Gunning S.J., Maggio F., Windley M.J., Valenzuela S.M., King G.F., Nicholson G.M. (2008). The Janus-faced atracotoxins are specific blockers of invertebrate KCa channels. FEBS J..

[85] Pineda S.S., Chin Y.K., Undheim E.A.B., Senff S., Mobli M., Dauly C., Escoubas P., Nicholson G.M., Kaas Q., Guo S. (2020). Structural venomics reveals evolution of a complex venom by duplication and diversification of an ancient peptide-encoding gene. Proc. Natl. Acad. Sci. USA.

[86] King G.F. (2019). Tying pest insects in knots: The deployment of spider-venom-derived knottins as bioinsecticides. Pest. Manag. Sci..

[87] Neto O.B.S., Valladão R., Coelho G.R., Dias R., Pimenta D.C., Lopes A.R. (2023). Spiders’ digestive system as a source of trypsin inhibitors: Functional activity of a member of atracotoxin structural family. Sci. Rep..

[88] Fuzita F.J., Pinkse M.W., Patane J.S., Verhaert P.D., Lopes A.R. (2016). High throughput techniques to reveal the molecular physiology and evolution of digestion in spiders. BMC Genom..

[89] Walter A., Bechsgaard J., Scavenius C., Dyrlund T.S., Sanggaard K.W., Enghild J.J., Bilde T. (2017). Characterisation of protein families in spider digestive fluids and their role in extra-oral digestion. BMC Genom..

[90] Valladão R., Coelho G.R., da Silva D.L., Neto E.B., Pimenta D.C., Chiariello T.M., Auada A.V., Wen F.H., Lopes A.R. (2020). Loxosceles gaucho venom gland proteome: A new perspective on *Loxosceles venom* biochemical composition. Toxicon Off. J. Int. Soc. Toxinol..

[91] Foradori M.J., Tillinghast E.K., Smith J.S., Townley M.A., Mooney R.E. (2006). Astacin family metallopeptidases and serine peptidase inhibitors in spider digestive fluid. Comp. Biochem. Physiol. B Biochem. Mol. Biol..

[92] Osteen J.D., Herzig V., Gilchrist J., Emrick J.J., Zhang C., Wang X., Castro J., Garcia-Caraballo S., Grundy L., Rychkov G.Y. (2016). Selective spider toxins reveal a role for the Nav1.1 channel in mechanical pain. Nature.

[93] Israel M.R., Tanaka B.S., Castro J., Thongyoo P., Robinson S.D., Zhao P., Deuis J.R., Craik D.J., Durek T., Brierley S.M. (2019). Na(V) 1.6 regulates excitability of mechanosensitive sensory neurons. J. Physiol..

[94] Casewell N.R., Wüster W., Vonk F.J., Harrison R.A., Fry B.G. (2013). Complex cocktails: The evolutionary novelty of venoms. Trends Ecol. Evol..

[95] Bohlen C.J., Priel A., Zhou S., King D., Siemens J., Julius D. (2010). A bivalent tarantula toxin activates the capsaicin receptor, TRPV1, by targeting the outer pore domain. Cell.

[96] Bohlen C.J., Chesler A.T., Sharif-Naeini R., Medzihradszky K.F., Zhou S., King D., Sánchez E.E., Burlingame A.L., Basbaum A.I., Julius D. (2011). A heteromeric Texas coral snake toxin targets acid-sensing ion channels to produce pain. Nature.

[97] Robinson S.D., Mueller A., Clayton D., Starobova H., Hamilton B.R., Payne R.J., Vetter I., King G.F., Undheim E.A.B. (2018). A comprehensive portrait of the venom of the giant red bull ant, *Myrmecia gulosa*, reveals a hyperdiverse hymenopteran toxin gene family. Sci. Adv..

[98] Deuis J.R., Zimmermann K., Romanovsky A.A., Possani L.D., Cabot P.J., Lewis R.J., Vetter I. (2013). An animal model of oxaliplatin-induced cold allodynia reveals a crucial role for Nav1.6 in peripheral pain pathways. Pain.

[99] Finol-Urdaneta R.K., Ziegman R., Dekan Z., McArthur J.R., Heitmann S., Luna-Ramirez K., Tae H.S., Mueller A., Starobova H., Chin Y.K. (2022). Multitarget nociceptor sensitization by a promiscuous peptide from the venom of the King Baboon spider. Proc. Natl. Acad. Sci. USA.

[100] Okada M., Corzo G., Romero-Perez G.A., Coronas F., Matsuda H., Possani L.D. (2015). A pore forming peptide from spider *Lachesana* sp. venom induced neuronal depolarization and pain. Biochim. Biophys. Acta.

[101] Zanchet E.M., Longo I., Cury Y. (2004). Involvement of spinal neurokinins, excitatory amino acids, proinflammatory cytokines, nitric oxide and prostanoids in pain facilitation induced by *Phoneutria nigriventer* spider venom. Brain Res..

[102] Little M.J., Wilson H., Zappia C., Cestèle S., Tyler M.I., Martin-Eauclaire M.F., Gordon D., Nicholson G.M. (1998). δ-atracotoxins from Australian funnel-web spiders compete with scorpion α-toxin binding on both rat brain and insect sodium channels. FEBS Lett..

[103] Little M.J., Zappia C., Gilles N., Connor M., Tyler M.I., Martin-Eauclaire M.F., Gordon D., Nicholson G.M. (1998). δ-Atracotoxins from australian funnel-web spiders compete with scorpion α-toxin binding but differentially modulate alkaloid toxin activation of voltage-gated sodium channels. J. Biol. Chem..

[104] Gilles N., Harrison G., Karbat I., Gurevitz M., Nicholson G.M., Gordon D. (2002). Variations in receptor site-3 on rat brain and insect sodium channels highlighted by binding of a funnel-web spider δ-atracotoxin. Eur. J. Biochem..

[105] Catterall W.A. (2012). Voltage-gated sodium channels at 60: Structure, function and pathophysiology. J. Physiol..

[106] Dib-Hajj S.D., Cummins T.R., Black J.A., Waxman S.G. (2010). Sodium channels in normal and pathological pain. Annu. Rev. Neurosci..

[107] Faber C.G., Lauria G., Merkies I.S., Cheng X., Han C., Ahn H.S., Persson A.K., Hoeijmakers J.G., Gerrits M.M., Pierro T. (2012). Gain-of-function Nav1.8 mutations in painful neuropathy. Proc. Natl. Acad. Sci. USA.

[108] Deuis J.R., Ragnarsson L., Robinson S.D., Dekan Z., Chan L., Jin A.H., Tran P., McMahon K.L., Li S., Wood J.N. (2021). The Tarantula Venom Peptide Eo1a Binds to the Domain II S3-S4 Extracellular Loop of Voltage-Gated Sodium Channel Na(V)1.8 to Enhance Activation. Front. Pharmacol..

[109] Rong M., Chen J., Tao H., Wu Y., Jiang P., Lu M., Su H., Chi Y., Cai T., Zhao L. (2011). Molecular basis of the tarantula toxin jingzhaotoxin-III (β-TRTX-Cj1α) interacting with voltage sensors in sodium channel subtype Nav1.5. FASEB J..

[110] Catterall W.A. (2018). Dravet Syndrome: A Sodium Channel Interneuronopathy. Curr. Opin. Physiol..

[111] De Jonghe P. (2011). Molecular genetics of Dravet syndrome. Dev. Med. Child. Neurol..

[112] Richards K.L., Milligan C.J., Richardson R.J., Jancovski N., Grunnet M., Jacobson L.H., Undheim E.A.B., Mobli M., Chow C.Y., Herzig V. (2018). Selective NaV1.1 activation rescues *Dravet syndrome* mice from seizures and premature death. Proc. Natl. Acad. Sci. USA.

[113] Xiao Z., Zhao P., Wu X., Kong X., Wang R., Liang S., Tang C., Liu Z. (2021). Variation of Two S3b Residues in KV4.1-4.3 Channels Underlies Their Different Modulations by Spider Toxin kappa-LhTx-1. Front. Pharmacol..

[114] Yuan C., Liao Z., Zeng X., Dai L., Kuang F., Liang S. (2007). Jingzhaotoxin-XII, a gating modifier specific for Kv4.1 channels. Toxicon.

[115] Yuan C., Yang S., Liao Z., Liang S. (2007). Effects and mechanism of Chinese tarantula toxins on the Kv2.1 potassium channels. Biochem. Biophys. Res. Commun..

[116] Ebbinghaus J., Legros C., Nolting A., Guette C., Celerier M.L., Pongs O., Bähring R. (2004). Modulation of Kv4.2 channels by a peptide isolated from the venom of the giant bird-eating tarantula Theraphosa leblondi. Toxicon.

[117] Gomes G.M., Dalmolin G.D., do Nascimento Cordeiro M., Gomez M.V., Ferreira J., Rubin M.A. (2013). The selective A-type K^+^ current blocker Tx3-1 isolated from the *Phoneutria nigriventer* venom enhances memory of naive and Aβ(25-35)-treated mice. Toxicon.

[118] Rigo F.K., Rossato M.F., Trevisan G., De Prá S.D., Ineu R.P., Duarte M.B., de Castro Junior C.J., Ferreira J., Gomez M.V. (2017). PhKv a toxin isolated from the spider venom induces antinociception by inhibition of cholinesterase activating cholinergic system. Scand. J. Pain..

[119] Almeida A.P., Andrade A.B., Ferreira A.J., Pires A.C., Damasceno D.D., Alves M.N., Gomes E.R., Kushmerick C., Lima R.F., Prado M.A. (2011). Antiarrhythmogenic effects of a neurotoxin from the spider *Phoneutria nigriventer*. Toxicon.

[120] Matsumura K., Yokogawa M., Osawa M. (2021). Peptide Toxins Targeting KV Channels. Handb. Exp. Pharmacol..

[121] Zamponi G.W., Striessnig J., Koschak A., Dolphin A.C. (2015). The Physiology, Pathology, and Pharmacology of Voltage-Gated Calcium Channels and Their Future Therapeutic Potential. Pharmacol. Rev..

[122] Nanou E., Catterall W.A. (2018). Calcium Channels, Synaptic Plasticity, and Neuropsychiatric Disease. Neuron.

[123] Vieira L.B., Kushmerick C., Hildebrand M.E., Garcia E., Stea A., Cordeiro M.N., Richardson M., Gomez M.V., Snutch T.P. (2005). Inhibition of high voltage-activated calcium channels by spider toxin PnTx3-6. J. Pharmacol. Exp. Ther..

[124] Tonello R., Fusi C., Materazzi S., Marone I.M., De Logu F., Benemei S., Gonçalves M.C., Coppi E., Castro-Junior C.J., Gomez M.V. (2017). The peptide Phα1β, from spider venom, acts as a TRPA1 channel antagonist with antinociceptive effects in mice. Br. J. Pharmacol..

[125] Rigo F.K., Trevisan G., Rosa F., Dalmolin G.D., Otuki M.F., Cueto A.P., de Castro Junior C.J., Romano-Silva M.A., do N. Cordeiro M., Richardson M. (2013). Spider peptide Phα1β induces analgesic effect in a model of cancer pain. Cancer Sci..

[126] Klint J.K., Berecki G., Durek T., Mobli M., Knapp O., King G.F., Adams D.J., Alewood P.F., Rash L.D. (2014). Isolation, synthesis and characterization of ω-TRTX-Cc1a, a novel tarantula venom peptide that selectively targets L-type Cav channels. Biochem. Pharmacol..

